# Cellular proliferation dynamics during regeneration in *Syllis malaquini* (Syllidae, Annelida)

**DOI:** 10.1186/s12983-021-00396-y

**Published:** 2021-05-27

**Authors:** Rannyele Passos Ribeiro, Bernhard Egger, Guillermo Ponz-Segrelles, M. Teresa Aguado

**Affiliations:** 1 Departamento de Biología, Facultad de Ciencias, Universidad Autónoma de Madrid, Madrid, Spain; 2 Institute of Zoology, University of Innsbruck, Innsbruck, Austria; 3 Animal Evolution & Biodiversity, Georg-August-Universität Göttingen, Göttingen, Germany

**Keywords:** Annelid, Dedifferentiation, Differentiation, Redifferentiation, Regeneration, Segmentation, Stem cells

## Abstract

**Background:**

In syllids (Annelida, Syllidae), the regenerative blastema was subject of many studies in the mid and late XX^th^ century. This work on syllid regeneration showed that the blastema is developed by a process of dedifferentiation of cells near the wound, followed by their proliferation and redifferentiation (cells differentiate to the original cell type) or, in some specific cases, transdifferentiation (cells differentiate to a cell type different from the original). Up to date, participation of stem cells or pre-existing proliferative cells in the blastema development has never been observed in syllids. This study provides the first comprehensive description of *Syllis malaquini’s* regenerative capacity, including data on the cellular proliferation dynamics by using an EdU/BrdU labelling approach, in order to trace proliferative cells (S-phase cells) present before and after operation.

**Results:**

*Syllis malaquini* can restore the anterior and posterior body from different cutting levels under experimental conditions, even from midbody fragments. Our results on cellular proliferation showed that S-phase cells present in the body before bisection do not significantly contribute to blastema development. However, in some specimens cut at the level of the proventricle, cells in S-phase located in the digestive tube before bisection participated in regeneration. Also, our results showed that nucleus shape allows to distinguish different types of blastemal cells as forming specific tissues. Additionally, simultaneous and sequential addition of segments seem to occur in anterior regeneration, while only sequential addition was observed in posterior regeneration. Remarkably, in contrast with previous studies in syllids, sexual reproduction was not induced during anterior regeneration of amputees lacking the proventricle, a foregut organ widely known to be involved in the stolonization control.

**Conclusions:**

Our findings led us to consider that although dedifferentiation and redifferentiation might be more common, proliferative cells present before injury can be involved in regenerative processes in syllids, at least in some cases. Also, we provide data for comparative studies on resegmentation as a process that differs between anterior and posterior regeneration; and on the controversial role of the proventricle in the reproduction of different syllid lineages.

**Supplementary Information:**

The online version contains supplementary material available at 10.1186/s12983-021-00396-y.

## Introduction

One of the early stages of body regeneration in annelids is the development of the blastema, a tissue composed by relatively undifferentiated cells that are able to proliferate [1–3]. The annelid blastema is considered to develop through a process of dedifferentiation [1, 4–6]. Dedifferentiation implies that already differentiated cells regress to a stemness state, and later redifferentiate (differentiate to the original cell type) or transdifferentiate (differentiate to a cell type different from the original) to restore lost tissues [1, 2, 4, 7]. However, annelid regeneration can additionally involve the participation of stem cells in some species [8, 9]. Migration of stem cells to the blastema was first documented for the sedentarian *Lumbriculus* by Randolph [8, 9] who named them neoblasts, a term now widely used to refer to the flatworm pluripotent stem cells [10], despite there being no evidence of homology of those cell types between annelids and flatworms. Evidence of migratory stem or germ cells to the blastema has been documented lately for other Sedentaria, such as *Phylo foetida* (Claparède, 1868) [11, 12], *Enchytraeus japonensis* Nakamura, 1993 [13–15], and *Capitella teleta* Blake, Grassle & Eckelbarguer, 2009 [16, 17]. However, only recently the first direct evidence of migration of a cell type similar to the one described by Randolph [8, 9] as neoblasts has been shown in the sedentarian *Pristina leidyi* Smith, 1896 [18, 19]. Out of Sedentaria, cells considered to be neoblasts have been reported for *Chaetopterus variopedatus* (Renier, 1804) [20] during posterior regeneration [21]. In addition, it has been suggested that a different type of stem cells might participate in intestine regeneration in the errantian *Platynereis dumerilii* Audouin & Milne Edwards, *1833* [22, 23].

During the 1960’s, a series of crucial studies by Boilly [24–34] described the cellular dynamics of anterior and posterior regeneration in Syllidae (Errantia). Engaged in the study of the origin of blastema cells in *Syllis amica* Quatrefages, 1866 [35], Boilly [27–29] observed that cells from the border of the wound participate in blastema development, and that no intersegmental migration of regenerative cells takes place when forming the blastema. With this evidence, Boilly [4] proposed a model in which the lineage of cells involved in blastema formation could vary depending on the part of the body being regenerated. In this model, regenerated musculature and coelomic epithelium would have a mesodermal origin (redifferentiation). Ectodermic cells would form nervous tissues, epidermis, the pharyngeal epithelium (redifferentiation), and, when regenerating posteriorly from the pharyngeal region, also the intestinal epithelium (transdifferentiation). Finally, endodermal cells would form the new intestine (redifferentiation). The model proposed by Boilly, together with evidence coming from other annelids, strengthened the hypothesis of regenerated tissues usually maintaining the germ layer identity from the pre-existing cells they originated from, i.e. ectoderm derives from ectoderm, endoderm derives from endoderm, and mesoderm derives from mesoderm [1, 4, 5, 36, 37]; with the exception of the intestine during posterior regeneration from the pharynx in Syllidae [32]. Interestingly, experiments specifically designed to reveal the dynamics of proliferative cells (S-phase cells) can be helpful to understand how blastema develops during regeneration, something that has not been done in syllids up to date.

Syllids exhibit a great variety of regenerative capacities that can be related to their different reproductive modes [38]. Although all syllid species known to regenerate can restore the posterior body, complete anterior regeneration is only known for the few species that show asexual reproduction by fission, as the Autolytinae *Procerastea halleziana* Malaquin, 1893 [39–42] and *Procearea picta* Ehlers, 1864 [41, 43, 44]; and the Syllinae *Syllis gracilis* Grube, 1840 [45–48], and *Syllis malaquini* Ribeiro et al., 2020 [49]. Notably, many studies have shown that the lack of proventricle during anterior regeneration can trigger sexual maturation by schizogamy (or stolonization), a process characterized by the induction of gonad development and gametogenesis, as well as metamorphosis of posterior ends (stolons). This relationship between anterior regeneration and sexual reproduction has been observed in *Syllis amica*, *Syllis prolifera* Krohn, 1852, and *Typosyllis antoni* Aguado et al., 2015 [38, 50–62], all of which are incapable of complete anterior regeneration. Here, we provide the first description of both anterior and posterior posttraumatic regeneration in *S. malaquini*, a syllid species that reproduces sexually by schizogamy and asexually by architomic fission [49]. Additionally, by tracking S-phase cells using thymidine analogues, we also describe the cellular proliferation dynamics throughout different stages of regeneration.

## Results

### General observations on the anatomy of* Syllis malaquini* and the distribution of S-phase cells

**Fig. 1:**
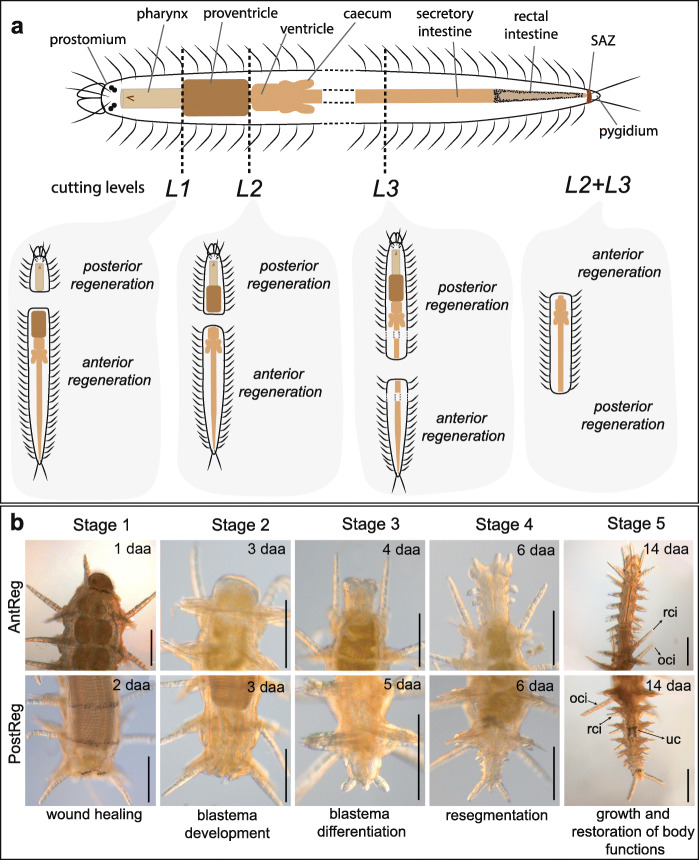
Experimental design and stages of regeneration. **a**. Anatomical characterization of *Syllis malaquini* and cutting levels of regeneration experiments: *L1*, after pharynx; *L2*, between proventricle and ventricle; *L3*, between segments 35–36; *L2 + L3*, bisections at *L2* and *L3* generating midbody fragments. Note that the end fragments were considered replicates of the experiments with cutting levels *L2* and *L3* (see Methods). **b**. Stages of anterior and posterior regeneration (see Methods); images taken from the experiment of the bisection at *L2*, as an example. Stage 3 is characterized by the appearance of the prostomium during anterior regeneration and the pygidium during posterior regeneration. Stage 5 is achieved when the restored digestive tube is completely differentiated and is functional. Scale bars: 200 μm. Abbreviations: **oci** original cirrus, **rci** regenerated cirrus, **uc** urinary concretions

The anatomy of *Syllis malaquini* is comparable to that of other syllids [39, 49, 59, 63] (Fig. 1a). The anterior region is characterized by a relatively cephalized prostomium (head, asegmental part) with eyes and antennae, and a peristomium (considered herein as the presetigerous segment following Heacox [64]) that bears two pairs of cirri. The body has a variable number of segments with cirri and parapodia that bear compound chaetae, and ends in a pygidium (tail, asegmental part). Running from anterior to posterior, the foregut of *Syllis malaquini* presents a tooth-bearing pharynx that extends to the first 6–8 segments; a proventricle, an organ that contains large muscular cells and two anterior plates, and that extends through 4–9 segments (see Fig. 4b in [49]); and a ventricle from which two ventricular caeca emerge (Fig. 1a). The intestine has two parts, recognized here by comparative anatomy as described for other syllids by Williams [65], Claparède [66] and Malaquin [39]. The anterior and medium portion of the intestine is identified as the glandular or secretory intestine (“*l’intestin glandulaire, secretant*”; sensu Malaquin [39]); and the posterior portion (last 7–9 segments of the body) is identified as the rectal intestine (“*l’intestin rectal ou urinaire*”; sensu Malaquin [39]) that contains urinary concretions (“*concrétions urinaires*”; sensu Malaquin [39]) located in two lateral grooves of the intestine walls. These urinary concretions can be seen as dark globules under the microscope (Fig. 1b/stage 5).

**Fig. 2:**
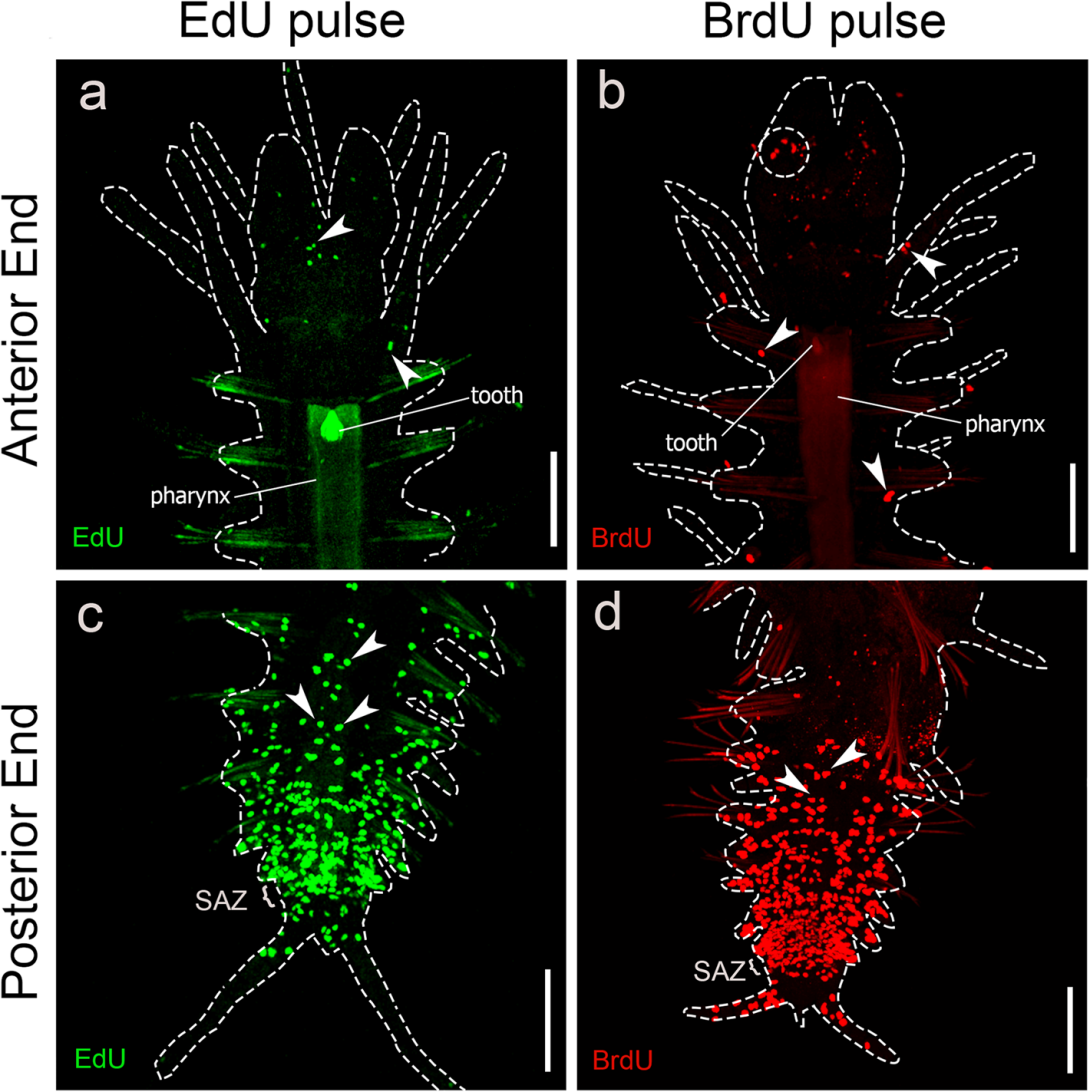
Distribution of S-phase cells in uncut specimens of *Syllis malquini*. **a**. EdU pulsed cells in anterior end. **b**. BrdU pulsed cells in anterior end, dashed circle indicates a region of the palp with an accumulation of S-phase cells. Arrowheads in (**a**) and (**b**) point to S-phase cells in the cirri. **c**. EdU pulsed cells in posterior end. **d**. BrdU pulsed cells in posterior end. Arrowheads in (**c**) and (**d**) point to S-phase cells located in the ventral midline. More details of S-phase cells in the posterior body and in the proventricle region can be seen in Additional files 1 and 2. Scale bars: 200 μm

Uncut animals were stained with EdU/BrdU-pulse to observe proliferating regions of *S*. *malaquini*’s body in non-experimental conditions (see Methods). Our results showed that S-phase cells are irregularly distributed in the anterior and midbody, being located in the prostomial appendages and dorsal parapodial cirri of anterior segments (Fig. 2a, b). Additionally, S-phase cells of the midbody were located in the ventral midline, cirri, and digestive tube (Additional files 1a–f and 2a–c). In contrast, the posterior body showed a prominent accumulation of S-phase cells in the growing segments and segment addition zone (SAZ, where new segments are generated [67]), with an overall average of 2.5x more S-phase cells than in the anterior body (*n* = 6 specimens). The observed animals presented a 2:7 anterior-to-posterior labelled cells ratio in the EdU-labelled specimens (*n* = 3), and 4:9 ratio in the BrdU-labelled specimens (n = 3) (Fig. 2c, d, Additional file 2a–c). Last, similar to what was observed at the anterior ends, S-phase cells were seen in the ventral midline of the posterior ends (Additional file 2b).

### Description of *Syllis malaquini* regeneration

In order to observe cell proliferation and blastema formation during regeneration, and the effect of proventricle absence in amputees of *S*. *malaquini*, we performed bisection-based experiments at different body levels: *L1*, *L2*, *L3* and *L2 + L3* (Fig. 1a; see Methods). In addition, in order to provide a more detailed description of our results, we divided the regeneration process in five developmental stages (Fig. 1b): 1) wound closure; 2) blastema development; 3) blastema differentiation, when the prostomium or pygidium appear; 4) resegmentation; 5) growth and restoration of body functions (the digestive tube is completely restored and the animals are able to feed). The mortality rate in our experiments was null.

#### Anterior regeneration

**Fig. 3:**
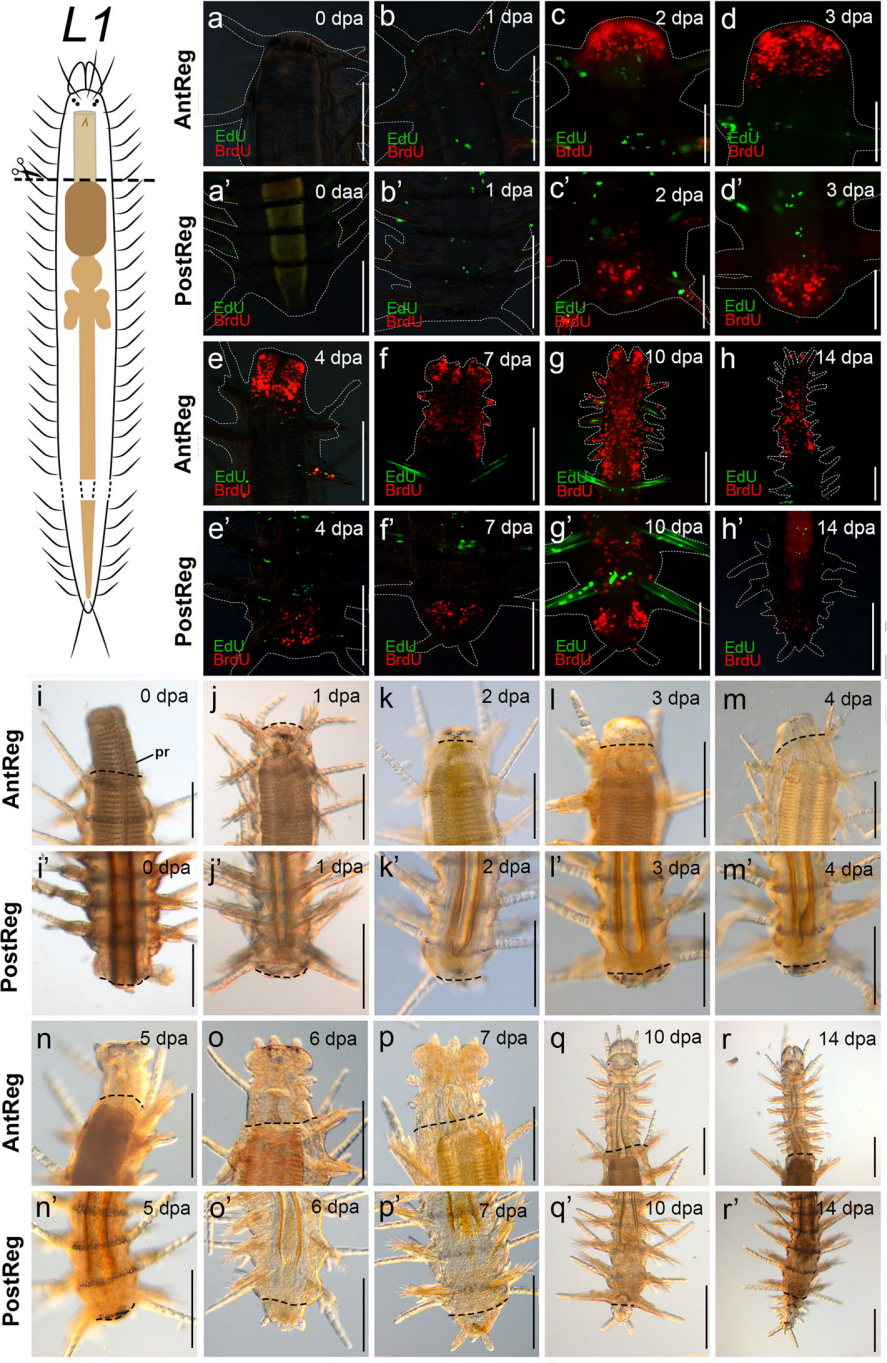
S-phase cell distribution and live observations and in regenerates, cutting level after pharynx (*L1*). **a**–**h**′. EdU (pulse-chase) BrdU (pulse) stainings. **a**–**h**. Anterior regeneration. **a’**–**h**′. Posterior regeneration. **i**–**r’**. Light microscopy images of living specimens. **i**–**r**. Anterior regeneration. **i**′–**r’**. Posterior regeneration. White dashed lines circumscribe the shape of the animals. Black dashed lines indicate amputation site. Abbreviation: **pr** proventricle. Scale bars: 100 μm (**d**, **d**′, **g’**), 200 μm (**a**–**c**, **e**–**h**, **a’**–**b**′, **e’**–**h**′, **i**–**r’**)

**Fig. 4:**
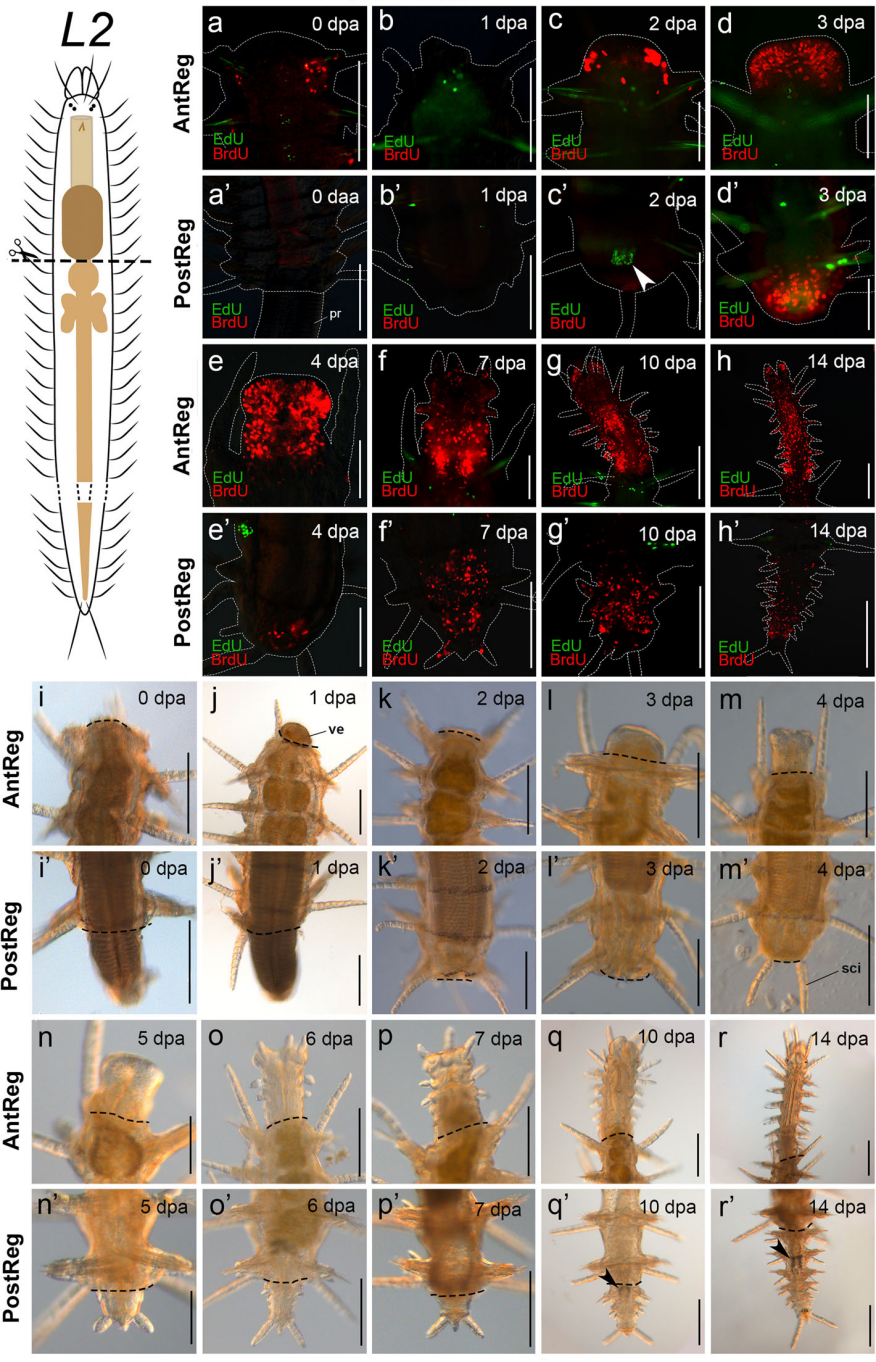
S-phase cell distribution and live observations and in regenerates, cutting level after proventricle (*L2*). **a**–**h**′. EdU (pulse-chase) BrdU (pulse) stainings. **a’**–**h**. Anterior regeneration. **ah**′. posterior regeneration; arrowhead in **c**′ points to EdU chased cells at the border of wounded foregut. **i**–**r’**. Light microscopy imaging of living specimens. **i**–**r**. Anterior regeneration. **i**′–**r’**. Posterior regeneration; arrowheads in **q’** and **r’** point to the region with urinary concretions in the rectal intestine. White dashed lines circumscribe the shape of the animals. Black dashed lines indicate amputation site. Abbreviations: **pr** proventricle **sci** dorsal cirri of the stock individual, **ve** part of ventricle protruding outward. Scale bars: 100 μm (**d**–**f**), 200 μm (**a**–**c**, **g**–**h**, **i**–**r’**)

**Fig. 5:**
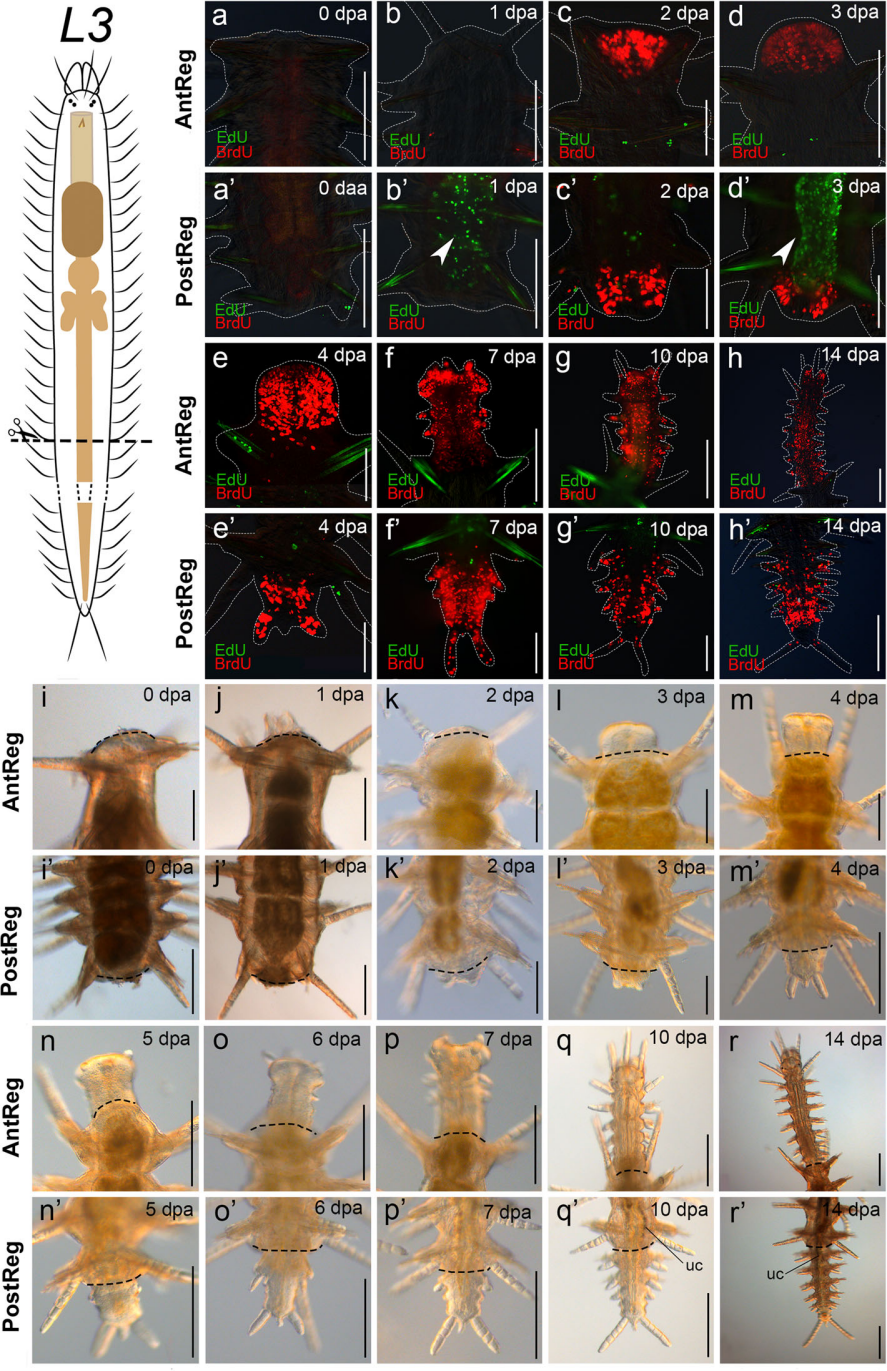
S-phase cell distribution and live observations and in regenerates, cutting level between segments 35 and 36 (*L3*). **a**–**h**′. EdU (pulse-chase) BrdU (pulse) stainings. **a**–**h**. Anterior regeneration. **e**. Confocal **a’**–**h**′. Posterior regeneration; arrowheads in **b**′ and **d**′ point to EdU chased cells in the stock gut. **i**–**r’**. Light microscopy imaging of living specimens. **i**–**r**. Anterior regeneration. **i**′–**r’**. Posterior regeneration; arrowheads in **q’** and **r’** point to the region with urinary concretions in the rectal intestine. White dashed lines circumscribe the shape of the animals. Black dashed lines indicate amputation site. Scale bars: 100 μm (**c**, **e**, **k**–**n**, **c**′–**e’**, **g’**, **k**′), 200 μm (**a**–**b**, **d**, **f**–**j**, **o**–**r**, **a’**, **b′**, **f**′, **h**′–**j’**, **l’**–**r’**)

**Fig. 6:**
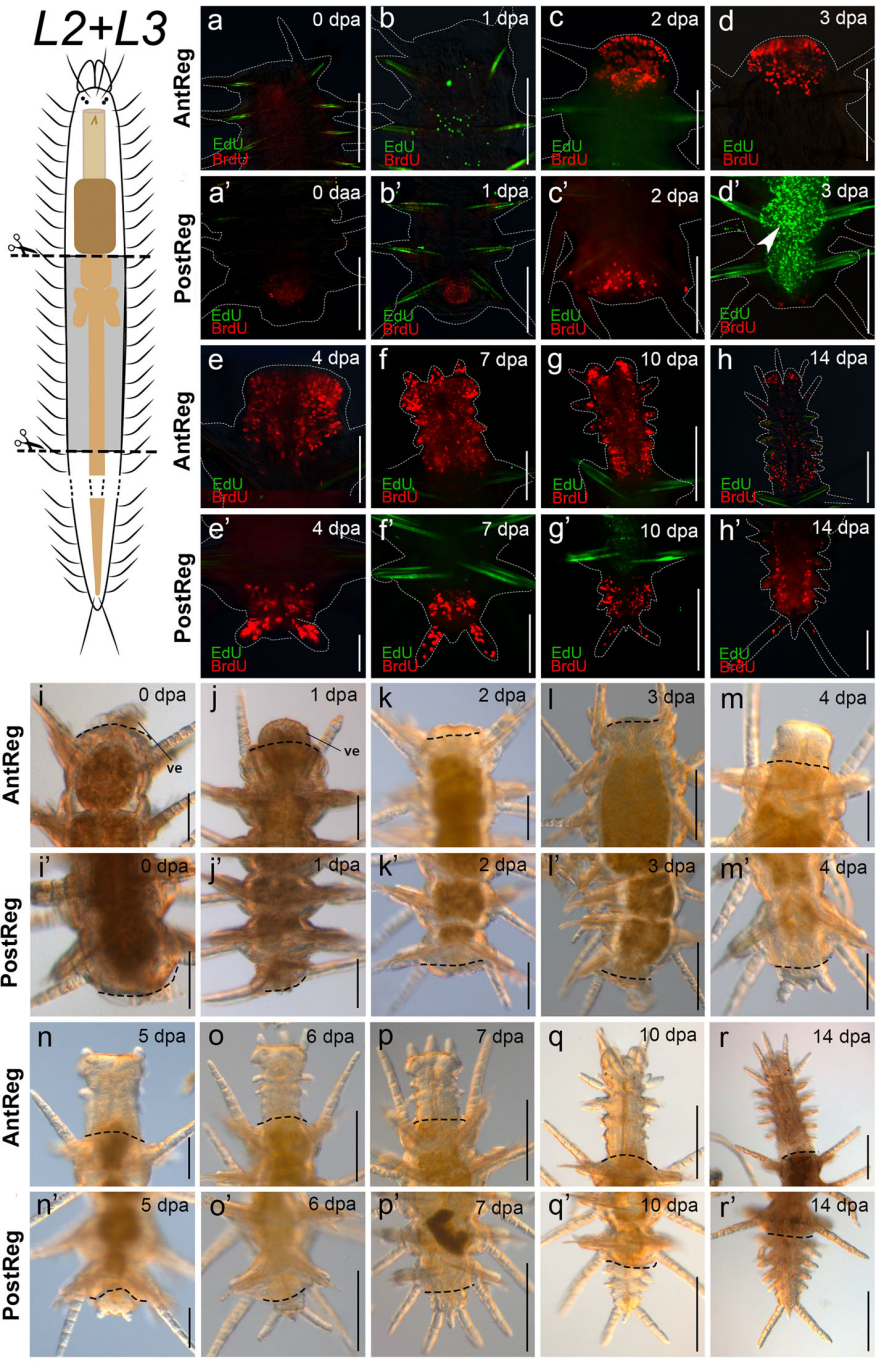
S-phase cell distribution and live observations and in regenerates, cutting level *L2* + *L3*, midbody fragments. **a–h**′. Edu (pulse-chase) BrdU (pulse) stainings. Fluorescence microscopy images of S-phase cell distribution in regenerates. **a**–**h**. Anterior regeneration. **a’**–**h′**. Posterior regeneration; arrowhead in **d**′ points to EdU chased cells in the stock gut. **i**–**r’**. Light microscopy images of living specimens. **i**–**r**. Anterior regeneration. **i**′–**r’**. Posterior regeneration. White dashed lines circumscribe the shape of the animals. Black dashed lines indicate amputation site. Abbreviation: **ve** part of ventricle protruding outward. Scale bars: 100 μm (**e**, **i**–**m**, **c′**, **e’**–**g’**), 200 μm (**a**–**d**, **f**–**h**, **n**–**r**, **a’**, **b′**, **d′**, **h′**–**r’**)

In all experiments, the specimens were able to completely restore the lost anterior body, although with few differences in pace (Figs. 3, 4, 5, and 6, Additional files 3 and 4). The main difference was that while in experiment *L1* specimens accomplished regeneration around 10–12 dpa, amputees of experiments *L2*, *L3* and *L2 + L3* only reached stage 5 after 14 dpa (days post-amputation).

**Fig. 7:**
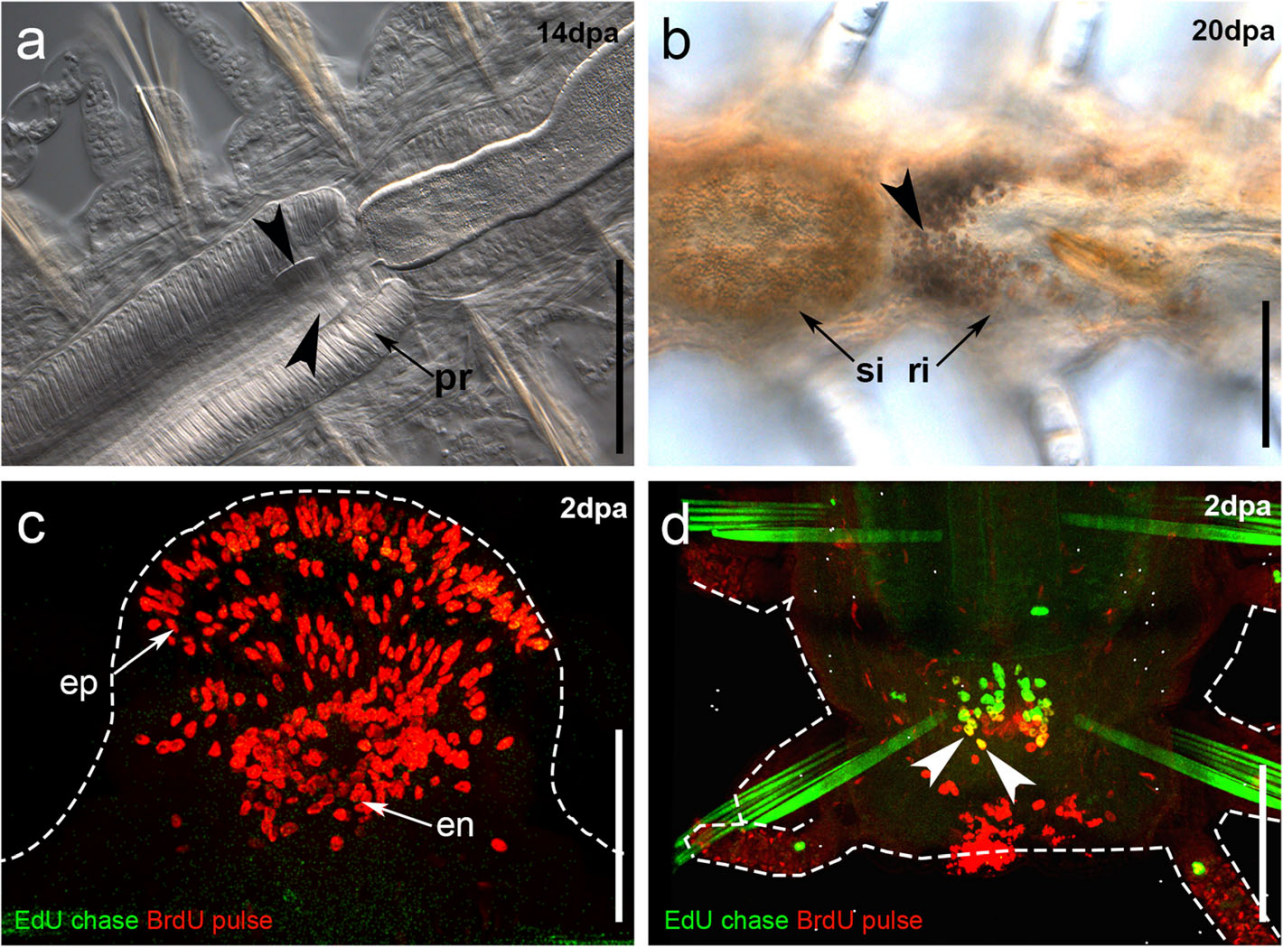
Details of stage 5 and blastema development of regenerating *Syllis malaquini*. **a**. Regenerated proventricle and pharynx (bisection at *L2* + *L3*); arrowheads point to proventricular plates on the anterior portion of proventricle. **b**. Regenerated posterior end (bisection at *L2*); arrowhead points to urinary concretions. **c**. S-phase cell distribution during anterior regeneration of an amputee cut at *L2* + *L3*, epidermal cells are on the border of the blastema, endodermal cells are centrally located in the blastema. **d**. Proliferative cells in the internal epithelium of the proventricle after bisection at *L2*, posterior regeneration. Arrowhead points to double-labelled cells (EdU-chased/BrdU-pulsed cells). **en** endodermal cells, **ep** epidermal cells. Dashed lines circumscribe the shape of the animals. Scale bars: 100 μm (**a**–**c**), 200 μm (**b**)

After bisection, the wound was closed by muscular contraction within two hours in specimens cut at *L1* (in which the proventricle usually protrudes outside the body and has to be retracted back inside; Fig. 3i), and immediately closed in specimens cut at *L2*, *L3* and *L2* + *L3* (Figs. 4i, 5i, 6 i). Somes specimens cut at *L2* and *L2+L3* showed the ventricle squeezed by the wound muscular contraction after 1 dpa (Fig. 4j, 6j). The wound was completely healed (stage 1) after 1–2 dpa in all experiments (Figs. 3j, 4k, 5j, 6k). Next, a blastema developed from 2–3 dpa (stage 2; Figs. 3k, l, 4 k, l, 5 k, l, 6 k, l; see also stainings in Figs. 3c, d, 4c, d, 5c, d, 6c, d). Stages 3 and 4 (blastema differentiation and resegmentation) started simultaneously after 4 dpa (*L2*, *L3*) or 5 dpa (*L1*, *L2* + *L3*) (Figs. 3m, n, Fig. 4m, n, 5m, n, 6m, n). Resegmentation seemed to slow down while amputees enlarged their newly generated appendages and differentiated the foregut. Once the mouth appeared (after 6–7 dpa), segment addition continued from the zone close to the amputation site with a clear anterior-to-posterior developmental gradient (around 10–14 dpa, Figs. 3o, p; 4o, p, 5o, r, 6o, r). When six to eight segments had been regenerated (around 10–14 dpa, Figs. 3q, r, 4q, r, 5q, r, 6q, r), segment addition was definitively interrupted. Then, amputees advanced to stage 5, when they enlarged the newly formed appendages and completed the differentiation of the digestive tube to make it functional. Amputees cut at *L1* regenerated a new pharynx around 10–12 dpa (Fig. 3q). Meanwhile, in the other experiments, the lost digestive organs were completely differentiated after 14–20 dpa. Amputees cut at *L2* regenerated the pharynx and the proventricle after 14 dpa (Fig. 4r). Amputees cut at level *L3* regenerated the pharynx, proventricle, and ventricle (with caeca) after 14 dpa (Fig. 5r). Amputees cut at *L2* + *L3* completed stage 5 after 15–20 dpa. Last, after 35 dpa, specimens of all experiments had almost reached the original body width (Additional file 3a–d). The regenerated pharynx and proventricle were morphologically similar to the original ones, even bearing the pharyngeal tooth and the proventricular plates (Fig. 7a). Thus, the examined specimens re-established digestive functions and were able to feed and grow again. By the end of the experiments, all specimens had been able to regrow six to eight new segments (*n* = 12, three specimens per experiment). No signs of stolonization were observed in the posterior end of any amputees at any time during the experiments.

#### Posterior regeneration

Posterior regeneration was characterized by the regeneration of the pygidium, SAZ, and the lost parts of the digestive tube. The regenerated segments arise from the SAZ and have an anterior-to-posterior developmental gradient. All specimens were able to regenerate the posterior body during the observed time of experimentation (35 dpa), although with some differences in the pace and extent of regeneration (Figs. 3, 4, 5, and 6, Additional files 3 and 4). In this case, despite amputees in experiments *L2*, *L3* and *L2 + L3* reaching stage 5 within 10–14 dpa, the wound closure (stage 2) in *L2* was delayed, probably because proventricle retraction was a prerequisite. Additionally, in specimens cut at *L1,* regeneration was delayed by at least 21 days, and resulted in the regrow of up to three segments, while up to eight segments were regenerated in the other experiments. This difference was probably due to the need to regenerate all post-pharyngeal digestive structures (proventricle, ventricle, secretory and rectal intestine). 

Specimens cut at *L1* stretched the segments close to the wound within 1–2 h after bisection (Fig. 3i’). Wound healing was completed after 1 dpa (stage 1, Fig. 3j’) and a small blastema developed during 2–5 dpa (stage 2, Figs. 3k’–n′; see also stainings in Figs. 3c’, d′). The pygidium appeared after 6 dpa (stage 3, Fig. 3o’). The anal opening was developed (7–10 dpa, Figs. 3p’–q’) and the SAZ was re-established within 10–14 dpa, when two segments were clearly visible (stage 4, Fig. 3r’). Stage 5 had been reached after 20 dpa, and the proventricle, ventricle, and intestine were completely differentiated only after 35 dpa. At this stage, the specimens had regenerated two or three segments, within which the proventricle and the intestine were squeezed (Additional file 3a’).

When animals were amputated at *L2*, the wound remained open with the proventricle protruding outside the body for some time (ranging from half an hour to more than 24 h) (Figs. 4i’, j’). After this time, the proventricle was retracted by muscular action. The wound was completely healed at 2 dpa (stage 1, Fig. 4k’) and a small blastema started to appear from 3–4 dpa (stage 2, Figs. 4l’, m’; see also stainings in Figs. 4d’, e’). After 5 dpa, the pygidium was regenerated (stage 3, Fig. 4n’). First signs of resegmentation were seen around 6–7 dpa (stage 4, Figs. 4o’, p′). However, segmentation was slightly delayed in some specimens as can be observed in Figs. 4p′. After 10–14 dpa, the rectal intestine was recognized by the presence of urinary concretions (stage 5, Fig. 4q’, r’). Finally, the animals regenerated up to eight segments after 35 dpa and the first regenerated segment (the closest to the amputation site) almost reached the width of the stock body segments (Additional file 3b’).

Specimens cut at *L3* closed the wound by muscular contraction immediately after bisection (Fig. 5i’), and completed stages 1 and 2 after 1 dpa (Fig. 5j’). The pygidium and two anal cirri could be distinguished at 2–3 dpa (stage 3, Fig. 5k’, l’). After 4 dpa, the amputees exhibited the first regenerated segment (stage 4, Fig. 5m’), and up to three segments were added in following days (stage 4, Figs. 5n’–p’). After 10–14 dpa, specimens reached stage 5, as recognized by the presence of urinary concretions in the rectal intestine (stage 5, Fig. 5q’, r‘). Stage 5 lasted at least until 35 dpa, when the animals had regenerated up to eight segments (Additional file 3C’).

After bisection, specimens cut at *L2* + *L3* close the wound by muscular contraction similarly to specimens cut at *L3* (Fig. 6i’). The amputees completed stage 1 after 1 dpa (Fig. 6j’). The blastema developed from 2–3 dpa, (stage 2, Fig. 6k’, l’; see also staining in Fig. 6c’) and the pygidium appeared after 4–6 dpa (stage 3, Fig. 6m’–o′). Resegmentation started at 7–8 dpa (stage 4, Fig. 6p’). Around 10–14 dpa, the animals had regenerated up to four segments (Fig. 6q’–r’) and the intestine was completely restored after 14–20 dpa (stage 5). By the last day of observation, the animals had regenerated up to seven segments (Additional file 3d’).

### Blastema development and cellular proliferation

In order to describe proliferation, S-phase cells were labelled using the thymidine analogues 5-ethynyl-2′-deoxyuridine (EdU) and 5-bromo-2′-deoxyuridine (BrdU). Those thymidine analogues are incorporated by annelid S-phase cells, as shown in previous studies [17, 23, 37, 68]. We used an EdU (pulse-chase)/Brdu (pulse) approach that allowed us to track cells that were in S-phase before amputation (labelled with EdU) and during regeneration (labelled with BrdU) [17, 69, 70].

#### Anterior regeneration

Despite the different cutting levels, cellular dynamics of S-phase cells during anterior regeneration were similar among all experiments (Figs. 3, 4, 5, and 6). An accumulation of BrdU pulsed cells on the border of the wound was first seen after 2 dpa, during blastema development (stage 2, Figs. 3c, 4c, 5c, 6c). S-phase cells labelled before bisection (EdU chased cells) were distributed in certain parts of the body of all amputees, mainly on the base of the dorsal cirri, epidermis, in the digestive tube, and in the posterior body. Interestingly, no EdU chased cells contributed to the development of the blastema; i.e. all S-phase cells in the blastema were BrdU pulsed ones, entering S-phase only after bisection (Figs. 3a–h, 4a–h, 5a–h, 6a–h and 7c). Following comparative data generated with other annelids and syllids, two different types of BrdU pulsed cells could be recognized in the blastema based on the shape of their nuclei [27, 28, 37]. Epidermal cells had elongated nuclei (Fig. 7c) and were located on the border of the blastema; endodermal cells had spherical nuclei (Fig. 7c) and were distributed in the inner blastema (gut region). Proliferation persisted at stage 5, during enlargement of the regenerates (14 dpa) (Figs. 3h, 4h, 5h, 6h).

#### Posterior regeneration

Similar to the anterior regeneration results, the blastema of posteriorly-regenerating individuals was seen as an accumulation of BrdU pulsed cells (Figs. 3a'–h', 4a'–h', 5a'–h', 6a'–h'). EdU chased cells were few and distributed in some parts of the dorsal cirri and in the ventral midline in amputees of all experiments. Notably, among all fixed posteriorly regenerating amputees (*n* = 96), double-labelled cells were only found in the digestive tube of two of the three amputees cut at *L2* (stage 2, 2 dpa, Fig. 7d), which means that cells labelled with EdU before cutting were proliferating during and at the site of regeneration. However, double labelled cells were not found in later stages of posterior regeneration at *L2*. Remarkably, 1:2 of specimens cut at *L3* and 1:7 of specimens cut at *L2 + L3* showed the original part of the intestine prominently occupied by EdU chased cells (Figs. 5b’, d′, 6d’), while the regenerated part was occupied only by BrdU pulsed ones (Figs. 5e’–h′, 6e’–h′). Finally, during stage 5 (10–14 dpa), amputees showed BrdU-labelled S-phase cells in the regenerated segments and in the SAZ (Figs. 3g’, h′, 4g’, h′, 5g’, h′, 6g’, h′).

## Discussion

### Overview of regeneration in *S*. *malaquini*

In this study, *Syllis malaquini* showed the ability to completely regenerate both the anterior and posterior body, even from midbody fragments, in agreement with previous study [49]. Anterior regeneration is accomplished when the prostomium, foregut, and about seven to nine segments are restored (within 14 dpa). Meanwhile, posterior regeneration is achieved when the pygidium, SAZ, and the lost parts of the digestive tube are restored and the individuals are capable of feeding and growing again, which occurred around 14 dpa for all experiments with the exception of specimens cut at *L1* (cutting after the pharynx). Posteriorly-regenerating amputees cut at *L1* regenerated a lower number of segments (e.g. only two segments at 14 dpa), but the proventricle and intestine were seen on the last day of observation (35 dpa), indicating a complete regeneration of the foregut and intestine (Additional file 3a'). Interestingly, complete anterior regeneration is also part of the life cycle of other syllid species that are able to reproduce asexually by architomy, for example, the ones in the clade of *S*. *malaquini– S. gracilis* (Syllinae) [39, 46, 49, 63] and in the group of *Procerastea–Proceraea* (Autolytinae) [40, 41]. Considering the phylogenetic hypotheses proposed for the whole family [61], this ability might be convergent in both groups.

### Blastema development

Our results on cellular dynamics showed that during both anterior and posterior regeneration, the blastema of all amputees (except two specimens cut at *L2*, 2dpa, Fig. 7d) was exclusively composed by BrdU pulsed cells, i.e. cells that entered S-phase after cutting. Thus, cells that were proliferating prior to cutting (EdU chased) did not significantly participate in the blastema development. Similar results have been described using EdU/BrdU techniques in the errantians *Parougia bermudensis* (Åkesson & Rice, 1992) [37, 71] and *Platynereis dumerilii* [23]. Interestingly, we identified putative endodermal and ectodermal proliferative cells by the shape of their nuclei following previous descriptions of those cells in the blastema formation of *P. bermudensis* [37] and *S. amica* [27–29], in which endodermal cells have spherical nuclei and ectodermal ones have elongated nuclei. Additionally, the distribution of these ectodermal and endodermal cells in the blastema was also similar to what has been described for *P. bermudensis* [37] and *S. amica* [27–29], i.e., cells with elongated nuclei peripherally distributed in the blastema and cells with spherical nuclei centrally located in the gut region (Fig. 7c). According to Boilly [27, 28], the cells involved in regeneration maintain their germ-layer identities, i.e. they redifferentiate. However, a detailed cell lineage study should be more suitable to corroborate this hypothesis. Notably, *S*. *malaquini* was able to regenerate posteriorly the intestine (endodermal origin [4]) from the foregut region, which has ectodermal and mesodermal origins (results of cut at *L1* and *L2*). This result probably indicates that, as previously suggested by Boilly, a process of transdifferentiation might take place when the regenerating fragment contains no endodermal tissues from which the gut can regrow [4, 32].

S-phase cells present prior to cutting (indicated by double staining) participated in the digestive tube regeneration of *S. malaquini* in posteriorly-regenerating amputees cut at *L2* (Fig. 5c’, Fig. 7 d). Notably, EdU signal might have decreased after several cell divisions and covered up by BrdU signal in later stages of regeneration in these specimens (Fig. 5f’–h). The foregut region was seen to contain S-phase cells in some uncut animals (Additional file 1a–f). Thus, the double-labelled cells observed in animals cut at *L2* were in S-phase in the proventricle region before bisection and contributed to restore the ventricle and intestine during posterior regeneration (Fig. 7d). Therefore, we might assume that the double-labelled cells observed in amputees cut at *L2* were probably restoring damaged tissues of the foregut as part of the regular homeostatic tissue repair before cutting (also observed in cirri and digestive tube of uncut animals, Fig. 2, Additional files 1 and 2), and later participated in regeneration. Among stem cells that can participate in blastema development, neoblasts have been described for annelids [8, 9]; however, we do not have enough data to infer whether the double-labelled cells observed in the digestive tube were indeed annelid neoblasts. In *Lumbriculus*, neoblasts were described to be located in the peritoneal epithelium of the ventral longitudinal muscles and to participate in the regeneration of mesodermal tissues [8, 9]. Here, as the double-labelled cells of *S*. *malaquini* were observed only in the internal epithelium of the proventricle, they might be a different type of proliferative cells rather than neoblasts. Taken together, these results might indicate that proliferative cells of the digestive tube probably involved in homeostasis can participate in regeneration, as has been suggested for the posterior regeneration of the annelid *Platynereis dumerilii* and other animals [23, 72]. On the other hand, the EdU chased cells observed in the intestine of posteriorly-regenerating amputees cut at *L3* (Fig. 4b’, e’) and *L2 + L3* (Fig. 5d’) were proliferating in the original part of the intestine before amputation. We did not observe participation of these cells in regeneration, as double-labelled cells were not identified.

As it is always the case with EdU-chase/BrdU-pulse studies, it is possible that some stem cells contributed to blastema formation, but were not in S-phase during the EdU treatment and were undetected consequently. Therefore, stem cells could participate in regeneration and still be undetected under two scenarios: either they have a slow cell cycle that diminished the likelihood of finding them in S-phase during EdU treatment; or they remain quiescent until regeneration is triggered. However, the use of several cutting levels in more than 120 specimens, together with the fact that we found no consistent clusters of BrdU stained cells that could be attributed to a stem-cell source other than the zone close to the blastema, make it difficult to associate our results with stem cell activity under either of these two scenarios.

### Resegmentation

As regards the stage of resegmentation, we noticed some differences between anterior and posterior regeneration in *Syllis malaquini*. Most annelids continue growing during their postembryonic development by action of a segment addition zone (SAZ), a growth region located in the posterior end that functions by sequential addition of segments [67, 73, 74]. The SAZ contains stem cells called teloblasts, which have been studied in detail in *Platynereis dumerilii* [73]. In this study, the SAZ of *S*. *malaquini* has shown to be permeated by proliferative cells in uncut animals and to be re-established during posterior regeneration for sequential addition of new segments. Two types of segment addition have been described for other animals (e.g. arthropods), simultaneous addition of segments, which occurs when the segments differentiate at the same time, and the sequential addition, in which segments are generated one at a time [75]. Interestingly, anterior regeneration in *S*. *malaquini* occurs through two different sequential events. The first one consists in the simultaneous development of the prostomium and the first two segments; while the second is a phase of sequential addition of segments that starts when the mouth appears and lasts until a maximum of 7–9 segments have been added. Similar observations have been previously reported for *Typosyllis antoni*, which shows simultaneous anterior addition of two or three segments in specimens cut after the proventricle and in specimens cut at the level of the intestine (without a proventricle); while sequential addition was seen when specimens were cut in front of the proventricle [59]. Meanwhile, sequential addition of segments has been proposed for *S. gracilis* and *P. halleziana* during anterior regeneration [40, 46]. Other annelids, such as *Timarete* cf. *punctata* Grube, 1859 [76, 77] and *Cirrineris* sp. [78], also show a first step of simultaneous addition followed by sequential addition of segments during anterior regeneration.

### Effect of proventricle absence in posterior ends

Interestingly, amputees of *S. malaquini* lacking the proventricle (*L2*, *L3*) did not stolonize in this study. This result contrasts with previous research, in which anteriorly-regenerating amputees that restore the prostomium and a proventricle-free foregut do stolonize, as observed in, for example, *S. amica* [29, 79, 80], *S. prolifera* [55, 56, 81, 82] and *T. antoni* [59, 62, 83]. The proventricle has been associated to a hormonal inhibition of the “stolonizing-promoting hormone” produced by the prostomium [55, 84]. Although studies on proventricle morphology showed no elements associated to endocrine functions [59, 85], data on gene expression indicate synthesis of hormones in the proventricle region (the whole body fragment containing the proventricle) that induce the production of sesquiterpenoids to regulate stolonization [86]. Thus, anterior-regenerating amputees that regenerate the prostomium and a proventricle-free foregut are supposed to stolonize. Interestingly, the anteriorly-regenerating amputees of *S. malaquini* lacking the proventricle did not stolonize in this study.

The differences between species might be interpreted in relation to the regenerative abilities and the capacity to reproduce asexually. *Syllis malaquini* and *S. gracilis* are able to reproduce asexually by architomy (a process of body fission followed by regeneration), implying the complete regeneration of the foregut in these species, including the proventricle [46, 53]; while there is no evidence for such a process in *S. prolifera, S. amica*, and *T. antoni* [53, 55, 56, 59, 60, 62, 81, 82]. Thus, assuming that the proventricle regulates stolonization, no effects would be expected when the proventricle can be regenerated, as in the case of *S*. *malaquini,* something that was already suggested by Durchon [53] for *S. gracilis*. However, studies including a broader taxonomic context and hormonal or genomic essays could clarify whether the proventricle is associated to a control of sexual reproduction in *S*. *malaquini* and other related species such as those of the *S. gracilis* species complex [49, 87].

## Conclusions

The regenerative blastema of *Syllis malaquini* develops exclusively from cells that enter S-phase after bisection, with the exception of the posterior regeneration from the level of the proventricle (*L2*), in which the participation of pre-existing S-phase cells was observed. Despite that dedifferentiation and redifferentiation are indicated as the processes that drive the blastema development, the possibility of stem cells participating in regeneration cannot be ruled out. In addition, we showed that simultaneous and sequential addition of segments seem to be involved in anterior regeneration, while only sequential addition takes place during posterior regeneration and regular growth. Interestingly, contrary to what has been shown for other syllids, anteriorly-regenerating amputees (those lacking the proventricle) did not stolonize in our experiments, differing from former studies that found stolonization as a response of absence of proventricle during anterior regeneration in other syllid species. This may be due to the *S*. *malaquini*'s ability to fully regenerate the proventricle.

## Methods

### Specimen culture and collection

Specimens of *Syllis malaquini* were obtained from aquaria of the University of Leipzig and maintained in the Institute of Zoology of the University of Innsbruck from March to May 2018 in two settings: in a marine aquarium and in Petri dishes with 3.5% artificial sea water (ASW), at room temperature (20–24 °C), feeding on TetraMin dry fish food flakes (Tetra GmbH).

### Experimental procedures

We selected 120 specimens with no signs of stolonization and with similar length (around 65 segments) for experiments. Four different cutting levels were used (see Fig. 2a): *L1*, bisection between pharynx and proventricle (anterior body fragment with eight or nine segments). *L2*, bisection between proventricle and ventricle (anterior body fragment usually with 14 or 15 segments). *L3*, bisection in the secretory intestinal region (between segments 34–35); and *L2* + *L3*, bisections at *L2* and *L3* were performed in the same individual resulting in a midbody fragment. The results of the regeneration of the prostomium and pygidium fragments in the operation at *L2* + *L3* were considered as replicates of those of posterior regeneration at *L2* and anterior regeneration at *L3*, and equivalent results were obtained (see Additional file 5). We bisected 30 specimens per experiment, which generated 60 amputees in *L1*, *L2* and *L3*, and 30 midbody amputees in *L2 + L3*. Amputees were maintained in Petri dishes with filtered ASW (3.5%), at room temperature, without feeding. They were allowed to regenerate during 35 dpa and observed daily under light microscopy.

### EdU (pulse-chase) BrdU (pulse) labelling

Six amputees per experiment (three for anterior and three for posterior regeneration) were fixed at each of eight different time points: 0 dpa, 1 dpa, 2 dpa, 3 dpa, 4 dpa, 7 dpa, 10 dpa, and 14 dpa (Additional file 5). As a result, 48 amputees were fixed per Edu/BrdU labelling experiment in the case of cuts at *L1*, *L2* and *L3*, and a total of 24 amputees were fixed in the case of cutting at *L2 + L3*. The remaining amputees were imaged and conserved in 70% ethanol at 20 and 35 dpa. We considered 14 dpa the last day for fixation because all identifiable regeneration stages could be observed by this time (Fig. 1b). Additionally, previous studies indicate that stolonization can occur from five days after amputation in anteriorly-regenerating amputees of *T. antoni* [59].

Complete living specimens were incubated in 0.04 mM EdU (Invitrogen, C10337) diluted in ASW for 60 min, at room temperature. Then, the animals were anesthetized in 7.14% MgCl_2_ hexahydrate for 10 min, and amputated at different cutting levels as described above (Fig. 1a). For each time point, the selected specimens were collected and incubated in 5 mM BrdU (Sigma B5002) diluted in ASW for 60 min (animals fixed for 0 dpa were treated with BrdU 60 min after amputation). Then, they were washed twice in filtered ASW, and relaxed in 7.14% MgCl_2_ hexahydrate for 10 min. Once relaxed, the specimens were fixed with 4% PFA in PBS for 60 min at room temperature. After fixation, the specimens were washed several times over a period of 30–60 min with PBS-T (1X PBS with 0.1% Triton X-100) and incubated in Protease XIV (0.1 mg/ml in PBS-T) at room temperature and under visual control for 30–90 min. DNA was denatured by incubation in 2 M HCl at 37 °C for 45 min. Then, the specimens were washed several times with PBS-T for 30–60 min, and rinsed in BSA-T (1% bovine serum albumin diluted in PBS-T) for 30 min. Next, they were incubated overnight in mouse-anti-BrdU (1:600 in BSA-T, Developmental Studies Hybridoma Bank G3G4) at 4 °C. Several washes were done with PBS-T (for about 30–60 min) to remove excess antibodies, followed by another 30 min incubation in BSA-T. For EdU staining, specimens were incubated for 2 h in freshly prepared EdU click-it reaction cocktail following manufacturer’s instructions (Click-iT™ EdU Alexa-488 kit, Invitrogen C10337). After EdU staining, the specimens were washed again several times with PBS-T for at least 30 min in dark conditions. Then, they were incubated for 60 min in the secondary antibody for BrdU staining, goat-anti-mouse (life technologies Alexa Fluor® 555 A-21422) 1:150 in BSA-T in dark conditions. Once the staining was completed, the specimens were rinsed several times in PBS-T during at least 30 min in dark conditions. Finally, the stained specimens were mounted on object slides with Vectashield (Vector Laboratories H1000). Positive and negative controls for EdU and BrdU stainings were done by omitting one of these thymidine analogues, and one of the BrdU antibodies.

Uncut control animals were labelled and stained using separate EdU pulse and BrdU pulse approaches in order to verify whether their incorporation was effective and to describe regions of proliferation in non-experimental conditions. For EdU pulse, the specimens were incubated in 0.04 mM EdU diluted in ASW for 60 min, at room temperature, anesthetized in 7.14% MgCl_2_ hexahydrate for 10 min, and subjected to the staining procedures of the Click-iT™ EdU Alexa-488 kit, following manufacturer’s instructions. BrdU pulsed specimens were fixed as described above with omission of the steps of EdU soaking and click-it reaction. S-phase cells were counted in anterior and posterior ends in three EdU/BrdU labelled specimens each (600 μm × 600 μm image area), using total confocal (BrdU)/fluorescence microscopy (EdU) projections.

### Microscopy and imaging

Light and fluorescence microscopy images for live and stained specimens were taken using a Leica DM 5000 B microscope (Leica, Germany) coupled with a Leica DFC 490 or Leica DFC 495 camera. All confocal stacks were generated on a Leica TCS SP5 II confocal Laser Scanning Microscope. Pictures were mounted using Imaris 9.2.

## Supplementary Information

**Additional file 1 **EdU pulse cross section and Z-projections of midbody in uncut specimens of *S*. *malaquini*. **a**. Ventral projection of pharynx region. B. Dorsal projection of pharynx region. **c**. Total projection of pharynx region. **d**. Ventral projection of proventricle region E. Dorsal projection of proventricle region F. Total projection of proventricle region. Abbreviations: **vmc** ventral midline S-phase cells, **fc** foregut S-phase cells. Scale bars: 100 μm.

**Additional file 2 **EdU pulse cross-sections of posterior end in uncut specimen of *S*. *malaquini*. **a**. Total Z-projection of posterior end. **b**. Ventral section. **c**. Dorsal section. Abbreviations: vmc ventral midline S-phase cells, **cic** cirri S-phase cell. Scale bars: 100 μm.

**Additional file 3 **Results obtained in the last day of observation (35 dpa). **a**–**d**, anterior regeneration. **a'**–**d'**, posterior regeneration. Thicker dashed lines indicate the bisection point. Thinner dashed lines circumscribe the proventricle. Abbreviations: **pr** proventricle, **ri** rectal intestine. Scale bars: 200 μm.

**Additional file 4 **S-phase cell distribution and live observations and in regenerates, cutting level *L2* + *L3*, end fragments. **a'**–**h'**. Edu (pulse-chase) BrdU (pulse) stainings. **a**–**h**. Anterior regeneration. **a'**–**h'**. Posterior regeneration. **i'**–**r'**. Light microscopy images of living specimens. **i**–**r**. Anterior regeneration. I′–R’. Posterior regeneration; arrowheads in Q’ and R’ point to the region with urinary concretions in the rectal intestine. Dashed lines circumscribe the shape of the animals. Scale bars: 100 μm (**e**, **i**–**m**, **c'**, **e'**–**g'**), 200 μm (**a**–**d**, **f**–**h**, **n**–**r**, **a'**, **b'**, **d'**, **h'**–**r'**).

**Additional file 5 **Setup of EdU (pulse-chase) BrdU (pulse) experiments performed with *Syllis malaquini*.

## Data Availability

The datasets supporting the conclusions of this article are included within the article and its additional files.

## Abbreviations

ASW: Artificial seawater
BrdU: 5-bromo-2′-deoxyuridine
BSA-T: 1% bovine serum albumin diluted in PBS-T
cic: Cirrius S-phase cell
dpa: Day(s) post amputation
en: Endodermal cell
EdU: 5-ethynyl-2′-deoxyuridine
ep: Epidermal cell
fc: Foregut S-phase cell(s)
L1: Level 1, bisection between pharynx and proventricle
L2: Level 2, bisection between proventricle and ventricle
L3: Level 3, bisection in the secretory intestinal region (between segments 34–35)
L2 + L3: Level 2 and level 3, simultaneous bisections at L2 and L3.
oci: Original cirrus
PBS: Phosphate buffer saline
PBS-T: 1X PBS with 0.1% Triton X-100
pr: Proventricle
rci: Regenerated cirrus
ri: Rectal intestine
sci: Dorsal cirri of the stock individual
uc: Urinary concretions
ve: Ventricle
vmc: Midventral line S-phase cells
