# Supplementary figures and images for: Cellular proliferation dynamics during regeneration in Syllis malaquini (Syllidae, Annelida)
Source: Front Zool. 2021 May 27;18:27. doi: 10.1186/s12983-021-00396-y (PMC8161976; doi:10.1186/s12983-021-00396-y)

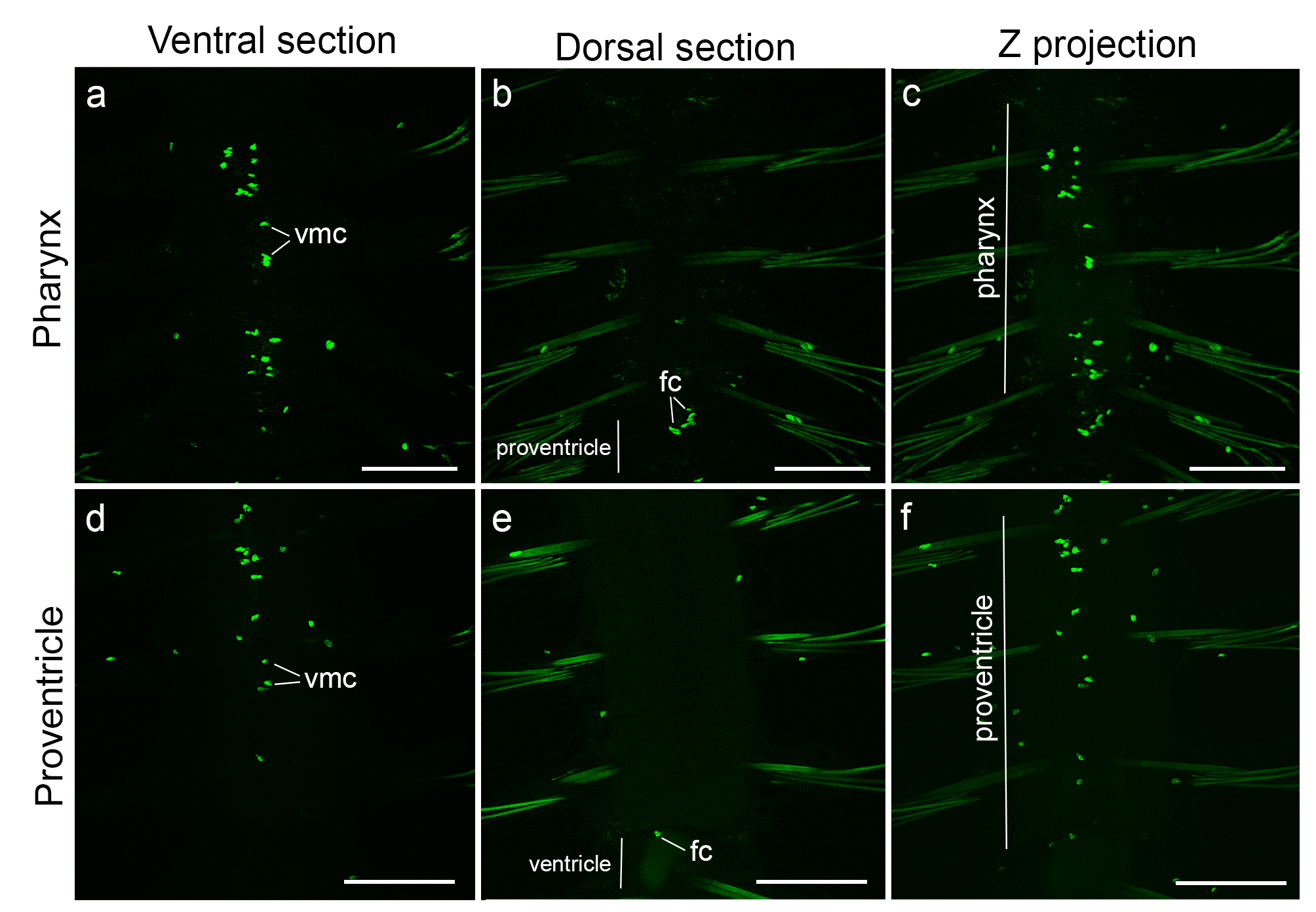

Supplement: Supplementary file 1 — Additional file 1 EdU pulse cross section and Z-projections of midbody in uncut specimens of S. malaquini. a. Ventral projection of pharynx region. B. Dorsal projection of pharynx region. c. Total projection of pharynx region. d. Ventral projection of proventricle region E. Dorsal projection of proventricle region F. Total projection of proventricle region. Abbreviations: vmc ventral midline S-phase cells, fc foregut S-phase cells. Scale bars: 100 μm. [file 12983_2021_396_MOESM1_ESM.tif]

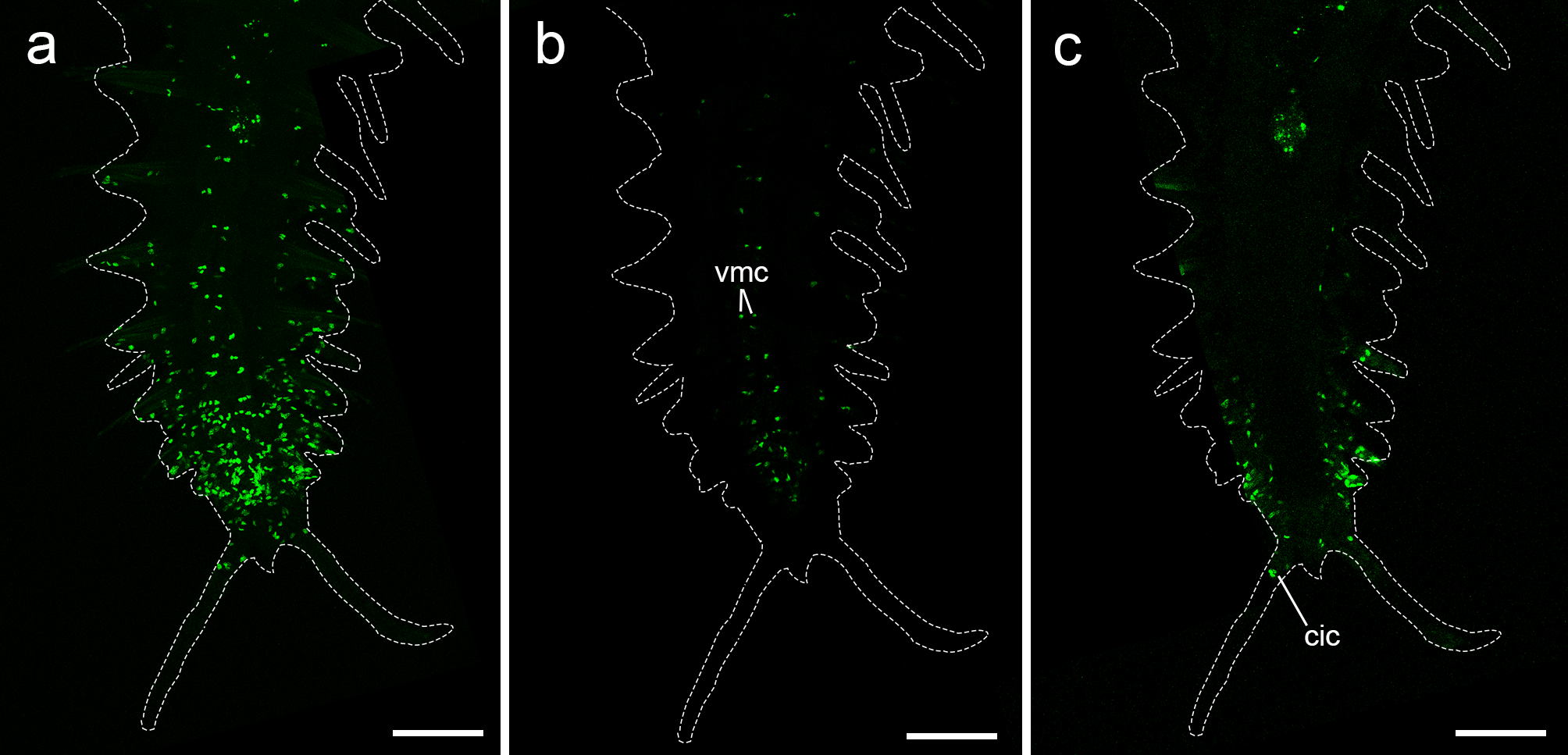

Supplement: Supplementary file 2 — Additional file 2 EdU pulse cross-sections of posterior end in uncut specimen of S. malaquini. a. Total Z-projection of posterior end. b. Ventral section. c. Dorsal section. Abbreviations: vmc ventral midline S-phase cells, cic cirri S-phase cell. Scale bars: 100 μm. [file 12983_2021_396_MOESM2_ESM.tif]

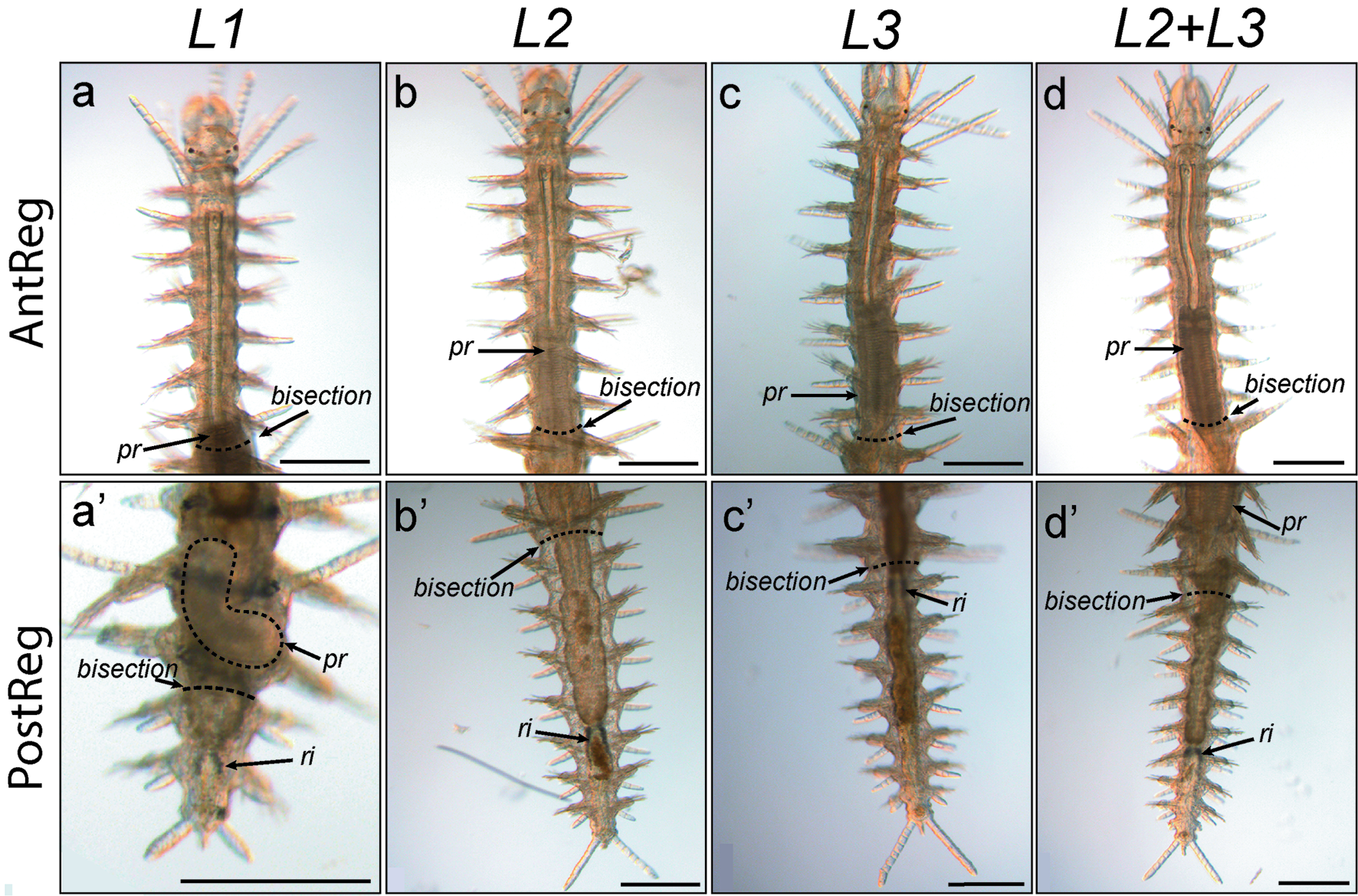

Supplement: Supplementary file 3 — Additional file 3 Results obtained in the last day of observation (35 dpa). a–d, anterior regeneration. a'–d', posterior regeneration. Thicker dashed lines indicate the bisection point. Thinner dashed lines circumscribe the proventricle. Abbreviations: pr proventricle, ri rectal intestine. Scale bars: 200 μm. [file 12983_2021_396_MOESM3_ESM.tif]

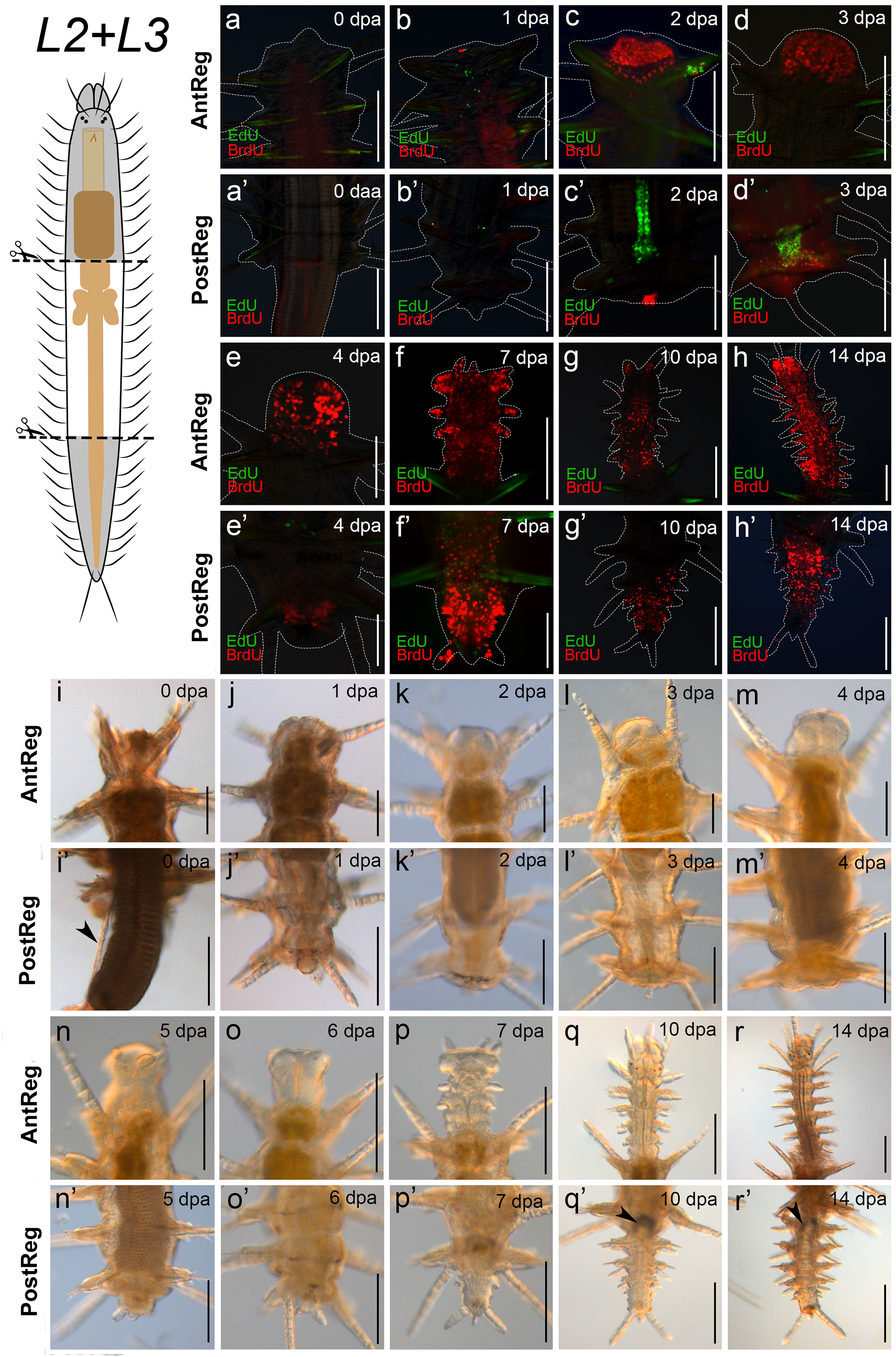

Supplement: Supplementary file 4 — Additional file 4 S-phase cell distribution and live observations and in regenerates, cutting level L2 + L3, end fragments. a'–h'. Edu (pulse-chase) BrdU (pulse) stainings. a–h. Anterior regeneration. a'–h'. Posterior regeneration. i'–r'. Light microscopy images of living specimens. i–r. Anterior regeneration. I′–R’. Posterior regeneration; arrowheads in Q’ and R’ point to the region with urinary concretions in the rectal intestine. Dashed lines circumscribe the shape of the animals. Scale bars: 100 μm (e, i–m, c', e'–g'), 200 μm (a–d, f–h, n–r, a', b', d', h'–r'). [file 12983_2021_396_MOESM4_ESM.tif]

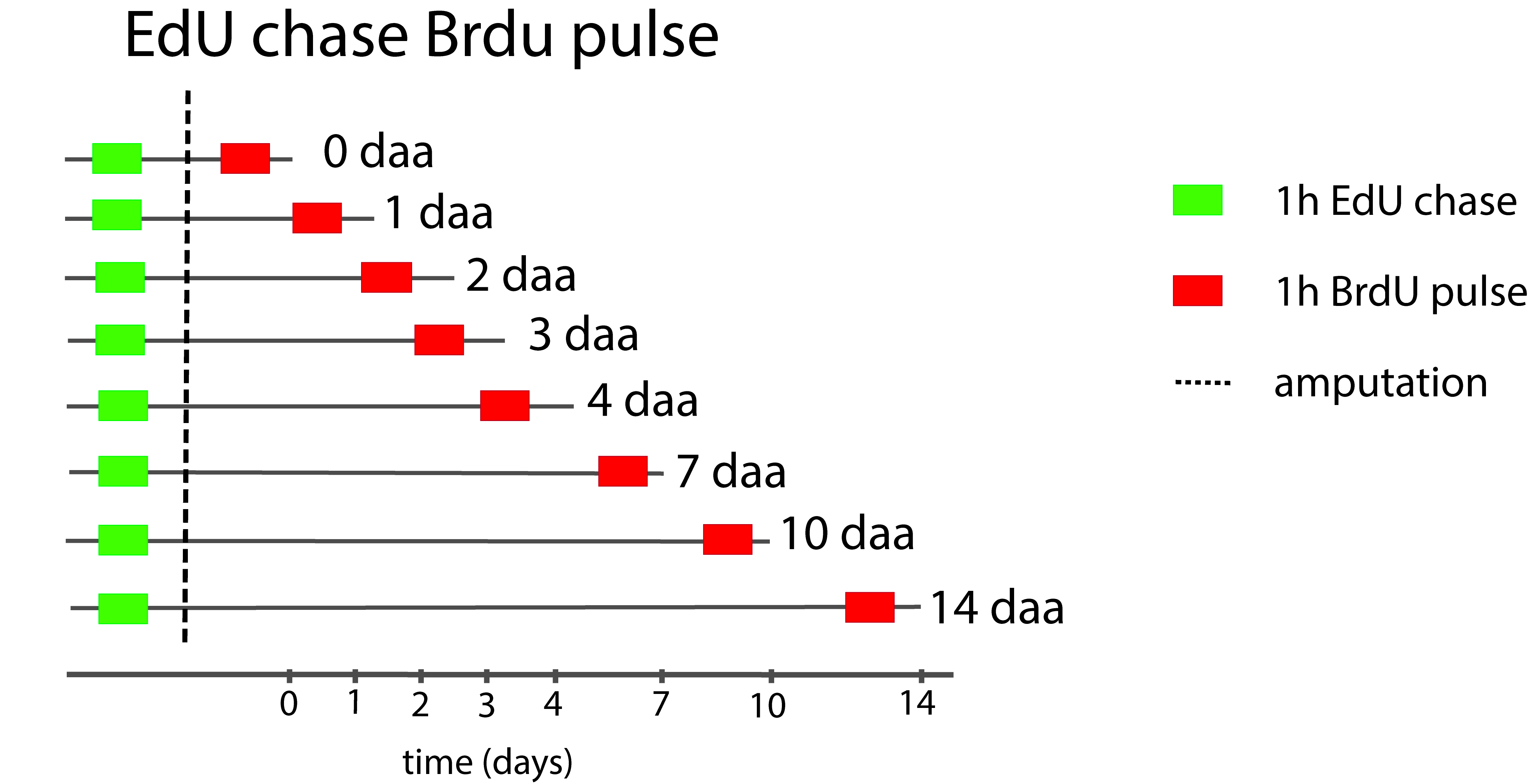

Supplement: Supplementary file 5 — Additional file 5 Setup of EdU (pulse-chase) BrdU (pulse) experiments performed with Syllis malaquini. [file 12983_2021_396_MOESM5_ESM.tif]
